# Soil carbon residence time regulates the age of dissolved organic matter in global rivers

**DOI:** 10.1093/nsr/nwag237

**Published:** 2026-04-21

**Authors:** Zhaohui Liu, Yongqiang Zhou, Gerard Rocher-Ros, Joshua F Dean, Jack J Middelburg, Pierre Regnier, Jan Karlsson, Liwei Zhang, Weipeng Lin, Chenglong Wang, Lei Zhou, Jianjun Wang, Yunlin Zhang, R Iestyn Woolway, Travis W Drake, Robert G M Spencer, Peter R Leavitt

**Affiliations:** Taihu Laboratory for Lake Ecosystem Research, State Key Laboratory of Lake and Watershed Science for Water Security, Nanjing Institute of Geography and Limnology, Chinese Academy of Sciences, Nanjing 211135, China; University of Chinese Academy of Sciences, Beijing 100049, China; Taihu Laboratory for Lake Ecosystem Research, State Key Laboratory of Lake and Watershed Science for Water Security, Nanjing Institute of Geography and Limnology, Chinese Academy of Sciences, Nanjing 211135, China; University of Chinese Academy of Sciences, Beijing 100049, China; Climate Impacts Research Centre (CIRC), Department of Ecology, Environment and Geoscience, Umeå University, Umeå 98107, Sweden; School of Geographical Sciences, University of Bristol, Bristol BS8 1SS, UK; Department of Earth Sciences, Utrecht University, Utrecht CS3584, The Netherlands; Biogeochemistry and Modelling of the Earth System-BGEOSYS, Department of Geoscience, Environment and Society, Université Libre de Bruxelles, Brussels 1050, Belgium; Climate Impacts Research Centre (CIRC), Department of Ecology, Environment and Geoscience, Umeå University, Umeå 98107, Sweden; State Key Laboratory of Estuarine and Coastal Research, East China Normal University, Shanghai 200062, China; Taihu Laboratory for Lake Ecosystem Research, State Key Laboratory of Lake and Watershed Science for Water Security, Nanjing Institute of Geography and Limnology, Chinese Academy of Sciences, Nanjing 211135, China; University of Chinese Academy of Sciences, Beijing 100049, China; School of Geography and Ocean Science, Ministry of Education Key Laboratory for Coast and Island Development, Nanjing University, Nanjing 210023, China; State Key Laboratory of Soil and Sustainable Agriculture, Institute of Soil Science, Chinese Academy of Sciences, Nanjing 211135, China; Taihu Laboratory for Lake Ecosystem Research, State Key Laboratory of Lake and Watershed Science for Water Security, Nanjing Institute of Geography and Limnology, Chinese Academy of Sciences, Nanjing 211135, China; University of Chinese Academy of Sciences, Beijing 100049, China; Taihu Laboratory for Lake Ecosystem Research, State Key Laboratory of Lake and Watershed Science for Water Security, Nanjing Institute of Geography and Limnology, Chinese Academy of Sciences, Nanjing 211135, China; University of Chinese Academy of Sciences, Beijing 100049, China; School of Ocean Sciences, Bangor University, Menai Bridge LL59 5AB, UK; Department of Environmental Systems Science, ETH Zürich, Zurich 8092, Switzerland; Department of Earth, Ocean and Atmospheric Science, Florida State University, Tallahassee, FL 32304, USA; Institute of Environmental Change and Society, University of Regina, Regina, Saskatchewan S4S 0A2, Canada

**Keywords:** dissolved organic carbon, river, radiocarbon, ancient carbon, soil organic carbon

## Abstract

Riverine dissolved organic carbon (DOC) constitutes a pivotal component in the Earth’s carbon cycle, yet little is known about the global patterns, sources, and factors governing lotic DOC. Here, we integrate a global dataset and employ machine learning to generate a global atlas of riverine DOC concentration and its radiocarbon (Δ^14^C) and stable-carbon (δ^13^C) isotopic signatures. Globally, riverine DOC has an average Δ^14^C value of –22.5‰ ± 144.0‰ (radiocarbon age of 221 years), with fossil carbon contributing a minor fraction (6.7% ± 3.0%). Terrestrial and autochthonous riverine production are the dominant DOC sources (>80%) at the global scale, with contemporary terrestrial DOC predominant in tropical rivers and within-river production prominent in those within temperate and semi-arid regions. Rivers draining high-latitude regions and high-elevation sites have the lowest Δ^14^C values (–353‰ to –78‰; ages between 3400 and 600 years). River Δ^14^C-DOC values correlate with soil organic carbon Δ^14^C values, but river DOC has much higher Δ^14^C values than subsurface soils indicating that riverine DOC originates from surface rather than subsurface soils. Because warming mobilizes aged organic carbon from permafrost soils, export to and processing of old carbon in recipient aquatic systems may accelerate with climate change.

## INTRODUCTION

Our conceptual understanding of rivers has evolved recently, shifting from passive ‘pipes’ transporting terrestrial carbon to dynamic ‘processors’ actively modifying carbon pools through storage, emission, and transformation [1–3]. Globally, rivers deliver 1.02 ± 0.22 PgC yr^–1^ to the ocean [4], while they, together with other inland waters, release 1.85 ± 0.50 PgC yr^–1^ to the atmosphere as carbon dioxide (CO_2_) and methane (CH_4_) [3–5]. Although most carbon in inland waters originates from terrestrial photosynthetic assimilation of atmospheric CO_2_ [2,3], it enters aquatic systems through multiple pathways. These include dissolved inorganic carbon (DIC) derived from soil respiration and weathering, dissolved and particulate organic carbon (DOC and POC) from terrestrial plants and soils, *in situ* aquatic primary production, and CH_4_ produced by microbes [1,6–8]. These pathways vary not only in carbon form but also in the timescales over which carbon is mobilized. While some DOC and POC may enter rivers within months to years of fixation, other pools—particularly DIC from carbonate weathering or DOC from deep soil layers—may reflect carbon that is centuries to millennia old [9–11]. Among organic carbon pools, DOC represents the dominant fraction in fluvial systems, comprising 63% of total organic carbon in global rivers [4] and up to 96% in some tropical rivers [12]. DOC plays crucial ecological roles by sustaining food webs [13], undergoing photochemical and microbial degradation to CO_2_ [14], impacting the underwater light regime [15], and regulating coastal and oceanic carbon cycling [16].

Riverine DOC sources are highly heterogeneous, encompassing terrestrial ecosystem exports, groundwater fluxes, and autochthonous aquatic production. The reactivity and biogeochemical fate of DOC are intrinsically linked to its source and chemical composition, with DOC from different sources exhibiting distinct ages and reactivity patterns that govern its mobilization dynamics and ecological functions [10,17]. Herein, we distinguish between modern carbon (<100 years, Δ^14^C > 0‰), aged carbon (centuries to millennia, Δ^14^C < 0‰ but > −500‰), ancient carbon (effectively ^14^C-free, Δ^14^C ≈ −950‰), and fossil carbon (petrogenic or combustion-derived ^14^C-free sources) [10,17,18]. While global-scale DOC studies have focused on concentrations and fluxes [4,5], information regarding its sources and ages remains limited. Fortunately, stable and radiocarbon isotope analyses of DOC (δ^13^C-DOC and Δ^14^C-DOC) are valuable tracers for discerning the sources and ages of carbon [6], providing a powerful approach for investigating riverine carbon biogeochemistry. While recent work has examined global riverine radiocarbon patterns in POC [17] and DIC [18] and revealed significant age variability, a comparable global assessment of the age and provenance of DOC—the dominant component of riverine organic carbon—is lacking.

River Δ^14^C-DOC signatures effectively trace carbon transfers between terrestrial and aquatic pools. In terrestrial ecosystems, vegetation-derived DOC exhibits positive (modern) Δ^14^C-DOC values reflecting atmospheric carbon fixed by photosynthesis, while petrogenic (rock-derived), peat, glacial, and permafrost-derived carbon sources all show negative (aged) Δ^14^C-DOC signatures [10,17]. Low Δ^14^C values reflect extended residence times of carbon in terrestrial landscapes, during which radioactive decay reduces ^14^C values. Consequently, soil-derived DOC exhibits variable Δ^14^C-DOC values depending on soil organic carbon (SOC) residence times and depth, as deeper horizons generally contain older carbon with longer turnover times and more negative Δ^14^C values [19]. In contrast, autochthonous aquatic DOC inherits its Δ^14^C signature from the DIC pool [11], and consequently may vary depending on the source and age of the DIC. Anthropogenic DOC (for example, sewage inputs) has multiple sources and may exhibit a negative Δ^14^C-DOC because of the presence of carbon derived from petrochemical-based products and urban wastes, as well as the presence of modern organic carbon compounds. Riverine Δ^14^C-DOC signatures should vary across continents, primarily depending on the time carbon spends within the landscape before reaching rivers. However, existing isotopic datasets of riverine DOC remain spatially limited and unevenly distributed, necessitating the need for comprehensive, high-resolution global coverage.

Evaluation of δ^13^C-DOC values provides complementary information that enables the differentiation of DOC from both terrestrial and aquatic sources and even distinguishes between different types of terrestrial plants. Such distinction is possible because δ^13^C values reflect differences in the inorganic carbon assimilated (atmospheric CO_2_ or DIC) and fractionation differences during photosynthesis [20]. For example, DOC from C3 plants (most terrestrial vegetation) has δ^13^C values ranging from –32‰ to –24‰ [21], C4 plants (for example, grasses) exhibit δ^13^C values of –13‰ to –10‰ [22], and aquatic autochthonous production (phytoplankton) typically fall between –23‰ and –17‰ [23]. δ^13^C-DOC signatures can further capture fractionation effects associated with microbial degradation and photochemical oxidation [20,24]. While analysis of Δ^14^C-DOC can be a powerful means of elucidating DOC dynamics and origins, it has limitations in fully resolving carbon sources when sources overlap in age. Combining δ^13^C with Δ^14^C analyses provides critical complementary information, enhancing source discrimination beyond what Δ^14^C alone can achieve.

**Figure 1.**
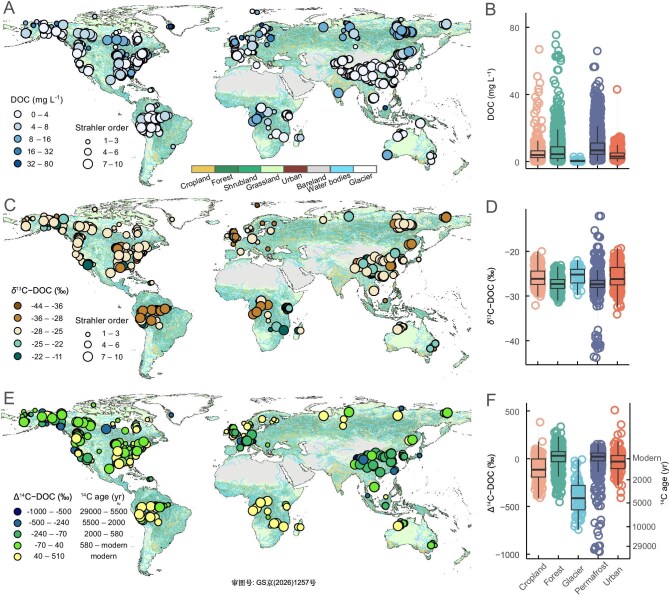
Global distribution of riverine DOC concentrations, δ^13^C values, and Δ^14^C values across different ecosystem types. Characteristics of (A, B) 5030 riverine DOC concentration samples from 2210 sites, (C, D) 2692 δ^13^C-DOC samples from 1051 sites, and (E, F) 2567 Δ^14^C-DOC samples from 1005 sites. The background map in panels (A), (C), and (E) depicts global land use in 2020. Panels (B), (D), and (F) present comparison of the different variables (DOC, δ^13^C-DOC, Δ^14^C-DOC) across various ecosystem types. In the boxplots, the horizontal line represents the median value, the bottom and top bounds of the boxes are first and third quartiles, and the whiskers represent the minimum and maximum values.

**Figure 2.**
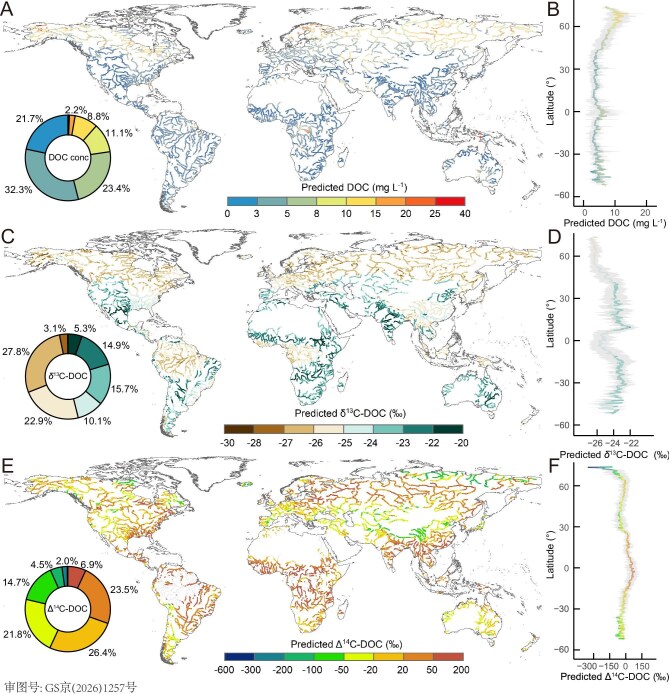
Global distribution and latitudinal patterns of predicted riverine DOC concentration, δ^13^C and Δ^14^C values. Predicted global patterns of riverine (A, B) DOC concentration, (C, D) δ^13^C-DOC, and (E, F) Δ^14^C-DOC. The map in panels (A), (C), and (E) illustrate major rivers as defined by the Global Runoff Data Centre (GRDC) database (https://mrb.grdc.bafg.de), incorporating 37 160 predicted data points. The colored lines in panels (B), (D), and (F) represent the mean predicted concentration, δ^13^C and Δ^14^C value of riverine DOC along latitudes (solid line), with shaded areas indicating ±1 standard deviation.

To elucidate the processes governing the nature of DOC (age, source) transported by rivers, we compiled an unprecedented global dataset, with 5030 DOC concentration measurements from 2210 sites, 2692 δ^13^C-DOC measurements from 1051 sites, and 2567 Δ^14^C-DOC measurements from 1005 river sites. We employed machine-learning models [17,25,26] to upscale site-level observations to global patterns of riverine DOC ages and provenance. The models showed strong agreement with observational data (Figs 1 and 2; Fig. S1), with the accuracy and spatial coverage of these maps match existing regional observations [10,11,27,28]. Model performance against withheld observations was robust for all parameters (Δ^14^C-DOC: *R*^2^ = 0.73, mean absolute error (MAE) = 44.38‰; DOC concentration: *R*^2^ = 0.71, MAE = 2.41 mg C L^−1^; δ^13^C-DOC: *R*^2^ = 0.62, MAE = 1.02‰; see Methods; Figs S2–S4). Given this performance, we used these high-resolution global estimates of riverine DOC concentration, δ^13^C, and Δ^14^C to quantify the main sources of DOC, identify the dominant factors governing DOC concentration and age, and evaluate the consequences of climate change on riverine DOC fluxes.

## RESULTS AND DISCUSSION

### Global patterns of riverine DOC concentration and its δ^13^C and Δ^14^C signatures

Riverine DOC concentrations vary three orders of magnitude globally, from below detection limits to 78.7 mg C L^–1^ and with a mean (±1 S.D.) of 6.6 ± 7.4 mg C L^–1^ (Fig. 1A and B). Permafrost-affected rivers exhibited the highest DOC (9.1 ± 7.9 mg C L^–1^), followed by forest-dominated (7.7 ± 10.2 mg C L^–1^), cropland-draining (5.7 ± 7.7 mg C L^–1^), and urban rivers (4.0 ± 3.5 mg C L^–1^), while glacial rivers have the lowest concentrations (0.5 ± 0.5 mg C L^–1^; Fig. 1B). Machine-learning models (Fig. 2A; Table S1) predict that >50% of global riverine DOC concentrations fall below 5 mg C L^–1^ (Fig. 2A), with a modest predictive uncertainty (1.9 ± 1.9 mg C L^–1^) (Fig. S1A and B). Latitudinally, riverine DOC concentrations peak in the Arctic-boreal region (6.9–14.2 mg C L^–1^) reflecting the combined effects of high carbon stocks in soils, extensive areas of permafrost-influenced peatlands, enhanced hydrological connectivity, and reduced in-stream mineralization under cold climate [29,30]. In contrast, tropical rivers show lower DOC concentrations, likely resulting from high terrestrial productivity, precipitation, and temperatures that enhance organic matter mineralization to CO_2_ and dilute export terrestrial DOC [4,12].

Observations of δ^13^C-DOC values showed a large variation globally (from –43.8‰ to –12.1‰, mean ± SD of –26.5 ± 2.7‰), signaling different sources of DOC (Fig. 1C). Tropical forest basins (for example, Amazon and Congo) consistently showed the lowest δ^13^C-DOC values (<–28‰), while certain Arctic rivers also exhibited exceptionally negative values (<–36‰). The low δ^13^C-DOC values in tropical systems aligns with the predominance of C3 vegetation (–32‰ to –24‰) [21], as well as intensive microbial processing of DOM during soil passage which can selectively remove ^13^C-enriched carbohydrates or proteins and leave ^13^C-depleted compounds such as lignin [24,31]. Alternatively, the extremely negative values observed in some tropical and Arctic rivers (<–30‰) may reflect inputs of organic C from deep-soil horizons where near-complete decomposition and microbial resynthesis produces ^13^C-depleted organic matter [19,32]. Finally, the extremely negative signatures (<–36‰) in some Arctic rivers may arise from methanogenesis in hypoxic environments and the subsequent incorporation of ^13^C-depleted methanotrophic biomass into exported terrestrial DOC [31]. In contrast to these low- and high-latitude sites, temperate rivers exhibit comparatively high δ^13^C-DOC values (–25‰ to –20‰), likely due to a mixture of C3 and C4 plant-derived carbon (–13‰ to –10‰), together with contributions from autochthonous aquatic primary producers [11,22]. Machine-learning model predictions reflected this marked latitudinal pattern in river δ^13^C-DOC, identifying the most negative values (–30‰ to –25‰) in both Arctic and tropical rivers (Fig. 2C and D) with a low predictive uncertainty (0.9‰ ± 0.4‰) (Fig. S1C and D).

Radiocarbon analysis shows that rivers carry DOC with ages ranging from freshly produced plant matter to carbon over 29 000 years old (from –974‰ to 509‰; mean ± SD: –22.5‰ ± 144.0‰; Fig. 1E). This wide variation is fundamentally linked to differences in DOM source; the oldest DOC (Δ^14^C < –800‰ corresponds to ^14^C ages > 13 000 years) originates from permafrost-affected streams in northeastern Siberia (for example, Kolyma River basin) and glacier-fed streams in southeastern Alaska, while forested systems exhibit predominantly modern carbon signatures (Fig. 1F). Aged DOC is consistently associated with soils disturbed by agriculture or forestry [19], mobilized peat deposits, thawing permafrost soils, retreating glaciers [23,27,28], and petrogenic compounds from agrochemicals and urban wastewater [33,34]. The machine-learning model exhibits a modest predictive uncertainty (18.8‰ ± 15.9‰) (Fig. S1E and F) and predicts that the most positive, modern values are found in wet tropical systems with high primary production (Amazon Basin, Congo Basin, and Southeast Asia). Conversely, the most negative Δ^14^C-DOC values reflect aged carbon sourced from Arctic and high-altitude systems (Alaska, Qinghai-Tibet Plateau, European Alps, Rocky Mountains, and Andes) (Fig. 2E and F). In general, predictions for data-sparse regions such as Africa and interior South America carry higher uncertainty (Fig. S1E and F) and require more cautious interpretation given limited spatial coverage (Fig. 1E).

**Figure 3.**
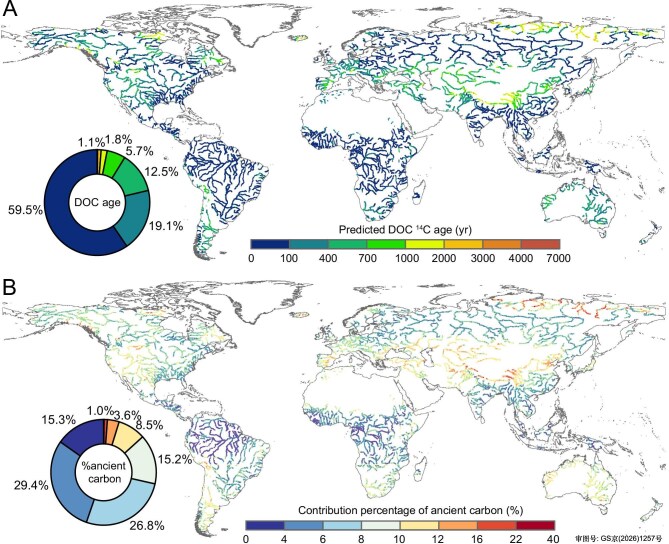
Global map of predicted ^14^C age of riverine DOC and calculated contribution percentage of ancient carbon to riverine DOC pool. (A) Predicted radiocarbon (^14^C) age of riverine DOC and (B) predicted percentage contribution of ancient carbon (^14^C ages > ∼55 000 years) to the riverine DOC pool. The map in panels (A) and (B) illustrate major rivers as defined by the GRDC database (https://mrb.grdc.bafg.de), incorporating 37 160 predicted data points (see ‘Methods’).

Overall, predicted ^14^C ages show that ∼60% of riverine DOC has ^14^C ages <100 years (Fig. 3A), indicating a predominantly modern origin derived from recent primary production. This youthful signature contrasts markedly with global riverine POC and DIC, where only ∼15% of POC [17] and ∼35% of DIC [18] have ^14^C ages <100 years. Similarly, only 3.1% of the global fluvial network has DOC with ^14^C ages exceeding 1000 years and is concentrated in high-latitude permafrost regions and high-altitude systems (Fig. 3A). This pattern is noteworthy, as these old-DOC rivers—particularly those draining northern latitudes—make important contributions to global river discharge, accounting for ∼10%–15% of total freshwater export to the ocean [35].

### ^14^C age and provenance of riverine DOC

High-resolution global maps of riverine δ^13^C-DOC and Δ^14^C-DOC were combined with a four-end-member isotopic mixing model to estimate the proportional contributions from modern terrestrial and autochthonous DOM (decadal, <100 years), aged Holocene deposits (millennial, hundreds to several thousands of years old), and ancient or fossil carbon sources (petrogenic, older than ∼55 000 years) to the global riverine DOC pool (Fig. 3B; Figs S5 and S6) [18]. Ancient carbon sources contribute only 6.7% ± 3.0% to global riverine DOC, reflecting the predominance of non-permafrost and fossil-free catchments. This estimate is broadly consistent with the 3.2%–8.9% range reported by Butman *et al.* [10] for ancient carbon contributions to riverine DOC based on watershed-scale studies in North America. However, the spatial variation in fossil carbon contributions to river DOC is high, ranging from negligible in most rivers up to 40% in arctic and high-altitude systems (Fig. 3B). The low riverine Δ^14^C-DOC signal in these regions is consistent with permafrost thaw and the erosion of older soils [29]. Regions with intensive agricultural activity (for example, central North America, Europe, and Japan) have moderate contributions of old carbon, likely reflecting shallower and younger soils affected by agricultural practices [34]. Globally, terrestrial organic carbon inputs and river autochthonous production contribute 38.2% ± 14.9% and 43.9% ± 14.8% to riverine DOC, respectively, and are most abundant in low- to mid-latitude rivers (Fig. S6A). Terrestrial DOC predominates in tropical river systems (for example, Amazon, Congo, and Southeast Asia rivers) but also represents a significant component in northern rivers, likely driven by primary production and mobilization of fresh organic matter during ice-free periods [36]. Autochthonous DOC is particularly important in temperate and semi-arid rivers, highlighting the role of aquatic primary production in sustaining DOC fluxes (Fig. S6B). In contrast, DOC derived from Holocene deposits (10.7% ± 4.5% of global riverine DOC) is concentrated in high-latitude floodplains (for example, Siberian lowlands, Mackenzie, and Yukon Rivers), where the reworking of organic-rich sediments—driven by processes such as permafrost thaw, peat degradation, and other hydrological disturbances—mobilizes aged carbon (Fig. S6C) [23]. Together, these patterns illustrate that, although ancient carbon sources are locally important, modern carbon from terrestrial and autochthonous production, together with aged Holocene carbon, are predominant in the global riverine DOC pool, with distinct geographical controls governing their distribution.

### Drivers of Δ^14^C in river DOC

**Figure 4.**
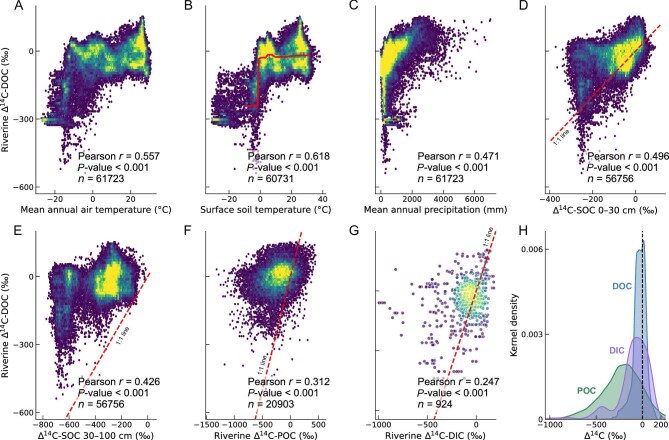
Relationships between riverine Δ^14^C-DOC and climatic variables, Δ^14^C-SOC, Δ^14^C-POC, and Δ^14^C-DIC. Pearson correlations between riverine Δ^14^C-DOC and (A) mean annual air temperature (MAT), (B) surface soil temperature, (C) mean annual precipitation (MAP), (D) Δ^14^C of surface soil organic carbon (SOC; 0–30 cm) [41], (E) Δ^14^C of subsurface SOC (30–100 cm) [41], (F) riverine Δ^14^C-particulate organic carbon (POC) [17], matched to Δ^14^C-DOC by geographic coordinates, and (G) riverine Δ^14^C-dissolved inorganic carbon (DIC) [18]. (H) A kernel density plot (*y*-axis represents the relative number of observations) of Δ^14^C of riverine DOC, POC, and DIC. The solid line in panel (B) represents the partial dependence of riverine Δ^14^C-DOC on surface soil temperature, derived from the machine learning model. The vertical dashed line in (H) shows the zero *x*-intercept. All correlations are derived from globally distributed data points and are statistically significant (*P* < 0.001).

Globally, climatic variables (for example, surface soil temperature, mean annual air temperature [MAT], mean annual precipitation [MAP], and climate water deficit) and soil properties (for example, silt and clay content, C/N ratio) emerge as important controls of the spatial distribution of Δ^14^C-DOC (Fig. S4B). Temperature has a strong impact on riverine Δ^14^C-DOC (Fig. 4A and B), particularly in permafrost landscapes where thawing likely couples soil carbon residence time with DOC export [23] (Fig. S4E, K). Water availability (MAP, climate water deficit, and evaporation) also substantially influences Δ^14^C-DOC (Fig. 4C; Fig. S4), presumably by enhancing leaching and lateral transport of both newly fixed and previously stored SOC. Collectively, temperature and hydrology regulate the global patterns of terrestrial ecosystem carbon turnover times [37], ultimately shaping the Δ^14^C signature of DOC exported to rivers. In addition to climate controls, we observed significant associations between Δ^14^C-DOC and hydrological variables, including surface and subsurface runoff (Fig. S7). Notably, the correlation with subsurface runoff suggests that groundwater flow exerts a substantial influence on riverine Δ^14^C-DOC, possibly in association with land-use changes and Δ^14^C-SOC signatures [19,32]. Subsurface runoff transports deeper Δ^14^C-depleted SOC to rivers, whereas land-use changes may alter SOC stocks and vertical distribution of carbon within soils, thereby modifying the Δ^14^C-SOC signatures of exported soil carbon. However, subsurface runoff integrates both shallow young interflow and deeper, ancient, potentially ^14^C-free, groundwater contributions, each with markedly different water and carbon residence times [38]. Further, groundwater can carry DOC that is substantially older than surface-derived sources due to prolonged subsurface residence times [39], potentially masking ancient DOC contributions in karst or deep aquifer settings.

Anthropogenic disturbances also shape riverine Δ^14^C-DOC variability, reflected by a high importance of population density in our model (Fig. S4). Anthropogenic impacts may either enrich or deplete ^14^C-DOC values. For example, impoundments can promote algal blooms [40], producing modern (^14^C-enriched) DOC. Conversely, urbanization and agriculture, particularly in rapidly developing regions of Asia and Africa, may export aged fossil carbon, including petroleum-based products and combustion-derived black carbon, contributing 3%–9% of riverine DOC [10]. DOC from tropical deforested and agricultural watersheds tends to be older (∼1500 ^14^C years) and more reactive than that from pristine environments [19], due to the exposure of deeper soil horizons resulting from agricultural expansion. Regionally, agricultural heartlands, such as those in the midwest U.S., Europe, and deforested eastern Congo, show moderate contributions of ancient carbon (10%–20%, Fig. 3B) due to deep plowing [19,34], while urban centers in East Asia exhibit variable signals reflecting both fossil fuel derived sewage [33] and impoundment induced algal production [40]. Together, these patterns highlight how regional land-use practices differentially shape DOC ages in rivers.

Soil properties are important variables for predicting riverine DOC source and age (Fig. S4B). A comparison of our global river Δ^14^C-DOC dataset with a published Δ^14^C-SOC product [41] shows significant correlations between riverine DOC and SOC, both in upper and deeper soil layers (Fig. 4D and E). This concurrence shows that the age of river DOC is predominantly controlled by the age of its major source, SOC. Interestingly, riverine DOC appears to originate primarily from surface soils (that is, leaching from topsoil) [30,42], because Δ^14^C-SOC values of surface SOC match those of riverine Δ^14^C-DOC, while deeper soils are substantially more negative. Surface soils increase near-surface DOC concentrations [30,42], which, together with higher hydrological connectivity, enhance leaching, whereas deeper soils retain older carbon via mineral sorption and more limited hydrologic connectivity [43]. Further, the spatial pattern of Δ^14^C-SOC is itself strongly influenced by MAT [41] such that surface soil temperature emerges as a key predictor of Δ^14^C-DOC in our global model (Fig. S4B). This association highlights the importance of soil carbon residence time regulating riverine carbon export and provides a physical basis for linking terrestrial carbon storage and mobilization to riverine carbon cycling.

Comparison of riverine Δ^14^C-DOC and Δ^14^C-POC [17] values reveals a moderate correlation (*r* = 0.31, *P* < 0.001; Fig. 4F) and shows that Δ^14^C-DOC is generally younger (mean ^14^C age: 221 years) than Δ^14^C-POC values in global rivers (mean ^14^C age of POC: 2649 years; Fig. 4H) [17]. This pattern suggests different paths of biogeochemical cycling for riverine POC and DOC pools. Particle mobilization requires soil erosion and is consistent with the importance of rainfall erosivity governing Δ^14^C-POC age patterns [17]. Moreover, POC transport involves multiple deposition-resuspension cycles, leading to substantial aging as organic matter is transported from land to downstream reaches [6,11,44]. In contrast, DOC mobilization is strongly governed by temperature, and its transport largely follows water flow paths, both on the surface and through shallow groundwaters (Fig. 4; Fig. S4). As such, we note that Δ^14^C-DOC variability is linked to differences in elevation and temperature (Fig. S4). Our analysis reveals that riverine DOC is slightly younger than surface soil SOC (mean Δ^14^C: –22.5‰ vs. –88.4‰; equivalent to a mean ^14^C age difference of ∼560 years) and much younger than subsurface soil SOC (mean Δ^14^C: –22.5‰ vs. –356.2‰; age difference ∼3350 years) and riverine POC (mean Δ^14^C: –22.5‰ vs. –226.3‰; age difference ∼2530 years) (Fig. 4D and F). This pattern indicates that carbon recently fixed by terrestrial and aquatic primary production predominates over aged soil and ancient carbon contributions to the DOC pool, consistent with the dual-isotope mixing model results (Fig. S6). The abundance of young DOC implies that riverine DOC is directly linked to changes in contemporary primary production on land and within inland waters. However, we also caution that our autochthonous end-member may not fully capture the isotopic variability of eutrophic temperate rivers, where different algal community composition (for example, cyanobacteria vs. green algae) can also affect δ^13^C values [45,46]. That said, Δ^14^C-DIC was weakly but significantly correlated with Δ^14^C-DOC (*r* = 0.25, *P* < 0.001; Fig. 4G), although DIC tends to be older (Fig. 4H) due to contributions from carbonate weathering, in-stream respiration of aged carbon pools, and groundwater sources—all processes which dilute terrestrial radiocarbon signals [18]. This correlation may reflect shared hydrological flow paths that simultaneously transport soil-derived DOC and DIC from terrestrial to aquatic systems [6,20], as well as in-stream production of DOC from DIC via aquatic photosynthesis [11], which can directly couple their isotopic signatures.

In summary, the integration of global maps of riverine DOC concentration, δ^13^C-DOC, and Δ^14^C-DOC reveals that most DOC is sourced from contemporary primary production and shows a close connection with surface SOC pools, reinforcing the idea that riverine DOC is largely exported from surface soils. However, the degree of predominance of surface-soil carbon sources also varies regionally: (i) lotic systems in lowland tropical and temperate basins exhibit predominantly modern DOC; (ii) rivers in permafrost regions have a high proportion of ancient DOC mobilized after thawing of frozen organic-rich soils [23,29]; and (iii) glacial systems can have substantial-aged DOC released from organic matter interbedded within degrading ice masses, as well as from atmospheric deposition of fossil-derived soot and black carbon [27,47,48]. However, because we did not distinguish between ice-entrained bio-labile organic matter [27] and bio-refractory fossil black carbon from the atmosphere [47], the ultimate fates of DOM released from glaciers cannot be resolved through this global analysis.

Changes in river DOC dynamics due to ongoing climate change should cascade to affect multiple biogeochemical and ecological processes in recipient aquatic ecosystems. Specifically, at high latitudes and high elevation regions, previously frozen aged soil carbon will be increasingly released. Some of this permafrost-derived DOC contains highly reactive organic matter, with up to 50% composed of low-molecular-weight organic acids which are readily consumed by microbial communities [49,50]. Thus, while radiocarbon age indicates time since fixation, it does not equate to recalcitrance; ancient permafrost and glacial DOC can be highly bioavailable [27,49], meaning its mineralization potential—not just its age—determines the strength of its potential feedback on climate. Further, as metabolic rates increase with rising temperature [51], the processing of this bioavailable and aged DOC may increase its mineralization to CO_2_ in rivers, particularly in the Arctic [7] and other high elevation settings [27,28,52,53]. These emissions may contribute to further planetary warming amplifying climate-carbon cycle feedbacks [54], while introducing ^14^C-depleted carbon into the atmosphere, analogous to fossil fuel combustion [10,19].

## MATERIALS AND METHODS

### Literature search and data extraction

A comprehensive database was compiled by searching major scientific databases, including Web of Science and Google Scholar, using the search terms: ‘DOC’ OR ‘dissolved organic carbon’ AND (‘C14’ OR ‘radiocarbon’ OR ‘age’ OR ‘young’ OR ‘old’) with cutoff date on 1st January 2025. In total, we compiled a global dataset derived from 197 peer-reviewed publications, consisting of 2567 Δ^14^C-DOC samples from 1005 rivers. In addition, 5030 riverine DOC concentrations from 2210 rivers and 2692 δ^13^C-DOC values from 1051 rivers were collected for further analysis. The compiled global river DOC dataset is available at Figshare with DOI: 10.6084/m9.figshare.30006 385.

Environmental variables employed for training of machine learning models and upscaling the Δ^14^C-DOC map were collected from published databases. These variables represent multiple aspects of Earth’s surface properties, including climate, soil properties, geomorphology, primary production, and potential anthropogenic disturbances (see Table S1 for a complete list and details). For global prediction and model training, all data were resampled to a uniform spatial resolution of 20 km.

### Feature selection and machine-learning model development

A global dataset comprising 5030 riverine DOC concentrations, 2692 δ^13^C-DOC values, and 2567 Δ^14^C-DOC observations was utilized to train machine-learning models. To develop robust models, we evaluated various machine-learning approaches, including linear regression, decision trees, k-nearest neighbors, extra trees, boosting and bagging methods, and random forest (see Supporting Information and Tables S2−S4 for full statistical model performance metrics). For each model, 70% of the observations were allocated for training, while the remaining 30% were reserved for independent testing. The optimized machine-learning models were subsequently used to generate the global distribution of riverine DOC concentrations, δ^13^C-DOC values, and Δ^14^C-DOC values (Figs S2–S4; Tables S2−S4).

### Isotopic mixing model and isotopic end-member determinations

The proportional contributions of four end-member sources—terrestrial, riverine autochthonous, Holocene deposits, and ancient (fossil) carbon—to the riverine DOC pool were estimated using an end-member mixing model tool (EMMTE). Four isotopically distinct end-member sources, including (i) terrestrial DOC, (ii) river autochthonous production, (iii) Holocene deposits, and (iv) ancient (fossil) carbon, were used in EMMTE (details in Supporting Information). We note that our comparison of instantaneous isotopic measurements with long-term climate averages potentially overlooks the effects of seasonal meteorological variability on DOC features [11,32]. Nonetheless, the robust global patterns recorded herein suggest that first-order climatic and edaphic controls predominate over seasonal variability as controls of riverine DOC age and provenance.

### Geospatial and statistical analyses

Details on geospatial analyses can be found in the Supporting Information. Statistical analyses, geospatial processing, and figure generation were performed using Python (v3.12.7), R (v4.7.1.2), and ArcGIS (v10.8). In all regression models, results with a *P*-value <0.05 were considered statistically significant.

## Supplementary Material

nwag237_Supplemental_File

## Data Availability

The data supporting our results are available in the Figshare repository (https://doi.org/10.6084/m9.figshare.30006385.v1). The codes supporting this study are available at (https://doi.org/10.6084/m9.figshare.30006385.v1).
