# Supplementary material for: Soil carbon residence time regulates the age of dissolved organic matter in global rivers
Source: Natl Sci Rev. 2026 Apr 21;13(9):nwag237. doi: 10.1093/nsr/nwag237 (PMC13198019; doi:10.1093/nsr/nwag237)
Supplement: nwag237_Supplemental_File [file nwag237_supplemental_file.docx]

**Supporting Information for**

Soil carbon residence time regulates the age of dissolved organic matter in global rivers

**Authors:**

Zhaohui Liu^1, 2, #^, Yongqiang Zhou^1, 2, *^, Gerard Rocher-Ros^3, #^, Joshua F. Dean^4^, Jack J. Middelburg^5^, Pierre Regnier^6^, Jan Karlsson^3^, Liwei Zhang^7^, Weipeng Lin^1, 2^, Chenglong Wang^8^, Lei Zhou^9^, Jianjun Wang^1, 2^, Yunlin Zhang^1, 2^, R. Iestyn Woolway^10^, Travis W. Drake^11^, Robert G.M. Spencer^12^, Peter R. Leavitt^13^

^1^Taihu Laboratory for Lake Ecosystem Research, State Key Laboratory of Lake and Watershed Science for Water Security, Nanjing Institute of Geography and Limnology, Chinese Academy of Sciences, Nanjing 211135, China

^2^University of Chinese Academy of Sciences, Beijing 100049, China

^3^Climate Impacts Research Centre (CIRC), Department of Ecology, Environment and Geoscience, Umeå University, Umeå 98107, Sweden

^4^School of Geographical Sciences, University of Bristol, Bristol BS8 1SS, UK

^5^Department of Earth Sciences, Utrecht University, 3584 CS Utrecht, the Netherlands

^6^Biogeochemistry and Modelling of the Earth System-BGEOSYS, Department of Geoscience, Environment and Society, Université Libre de Bruxelles, 1050 Brussels, Belgium

^7^State Key Laboratory of Estuarine and Coastal Research, East China Normal University, Shanghai 200062, China

^8^School of Geography and Ocean Science, Ministry of Education Key Laboratory for Coast and Island Development, Nanjing University, Nanjing 210023, China

^9^State Key Laboratory of Soil and Sustainable Agriculture, Institute of Soil Science, Chinese Academy of Sciences, Nanjing 211135, China

^10^School of Ocean Sciences, Bangor University, Menai Bridge LL59 5AB, Anglesey, Wales, UK

^11^Department of Environmental Systems Science, ETH Zürich, Zurich 8092, Switzerland

^12^Department of Earth, Ocean and Atmospheric Science, Florida State University, Tallahassee 32304, USA

^13^Institute of Environmental Change and Society, University of Regina, Regina, Saskatchewan S4S 0A2, Canada

# Zhaohui Liu and Gerard Rocher-Ros contributed equally to this work

*Correspondence: [yqzhou@niglas.ac.cn](mailto:yqzhou@niglas.ac.cn) (Y. Zhou)

*Processing of compiled dataset*

When latitude and longitude were not reported in the literature, they were determined using Google Maps (<http://www.google.cn/maps>) based on the descriptions of study sites. Radiocarbon values reported as fraction modern (F_m_) or percent modern (pM) were converted to Δ^14^C values and subsequently to ^14^C age using the following equations:

$F_{m}=0.01\times pM$ (1)

$\Delta^{14}C=1,000\times[{F_{m}\exp}^{-\lambda\left( y-1950 \right)}-1]$ (2)

${}^{14}C age (yr)= -8,033 ln(F_{m})$ (3)

where 1950 is the standard radiocarbon reference year (pre-bomb atmospheric testing baseline) [1], $\lambda$ = 1/8,267 yr^–1^ is the decay constant based on the true half-life of ^14^C, 8,033 is derived from the Libby half-life of ^14^C [1], and y represents the year of sample collection.

Climatic factors, which primarily control biospheric carbon turnover, are considered potential drivers of Δ^14^C-DOC values [2, 3], including mean annual temperature, surface soil temperature, mean annual precipitation, climate water deficit, reference evapotranspiration, total evaporation, and vapor pressure deficit. Soil properties, which affect the preservation and microbial decomposition of organic carbon, and may thus influence riverine DOC concentrations and Δ^14^C-DOC values [4]. These variables include surface volumetric soil water (0−7 cm depth, representing near-surface saturation), soil moisture (derived from a one-dimensional soil water balance model, reflecting integrated root zone water availability), the Palmer Drought Severity Index, carbon-to-nitrogen (C/N) ratio, available water content, sand, silt, and clay content. Geomorphological factors that influence the transport and storage of DOC in river systems [5] include elevation and slope. Primary productivity variables, which represent processes like photosynthesis and respiration, have been shown to significantly affect riverine Δ^14^C-DOC values[6], including gross primary productivity (GPP), net primary productivity (NPP), and total soil respiration. Anthropogenic disturbances, such as land use conversion and fossil fuels combustion, have the potential to reintroduce depleted ^14^C signal to riverine Δ^14^C-DOC [3, 7, 8]. Relevant anthropogenic variables include population density, average DNB radiance (nighttime light intensity), and the human development index (HDI). Supplementary Table S1, and Supplementary Fig. S2–S4 provide detailed descriptions, data sources, and interrelationships with all environmental variables.

### *Feature selection and machine-learning model development*

First, we excluded independent variables exhibiting strong multicollinearity (Pearson’s *r* > 0.95) to reduce redundancy. An initial set of 27 environmental variables were selected to develop models. However, an excessive number of input variables can increase model complexity and uncertainty [9]. To determine the optimal subset of explanatory variables for efficient modeling, Pearson correlation analysis and random forest-based feature importance (quantified by %IncMSE) were applied. Specifically, Pearson correlation coefficients between each variable and Δ^14^C-DOC were calculated, and feature significance was evaluated using the “randomforest” package in R (version 4.7.1.2). The *p*-values of the variable were obtained through permutation testing. To obtain the optimal combination of independent variables, recursive feature elimination (RFE) was performed using 5-fold cross-validation on the training set to screen out unimportant variables, combined with the results of correlation coefficients and feature importance metrics. The optimal variable subset was selected based on the highest cross-validated accuracy (lowest RMSE) across folds. Model performance peaked when 10 variables were included, yielding the lowest RMSE. As a result, 10 environmental variables were selected for Δ^14^C-DOC machine-learning model development: surface soil temperature, surface soil carbon/nitrogen (C/N) ratio, silt and clay fraction, climate water deficit, total evaporation, mean annual temperature, mean annual precipitation, gross primary production, elevation, and the Human Development Index (HDI). The same feature selection methodology was applied independently to the models for riverine DOC concentrations and δ^13^C-DOC values. Different sets of environmental variables were used for these models. The optimal variables for riverine DOC concentration include available water content, *R* factor of rainfall erosivity, soil loss, surface soil carbon/nitrogen (C/N) ratio, elevation, population density, climate water deficit, surface runoff sum and gross primary productivity. The optimal variables for riverine δ^13^C-DOC values include climate water deficit, palmer drought severity index, reference evapotranspiration, surface shortwave radiation, vapor pressure deficit, silt and clay fraction, slope, elevation, mean annual temperature and net primary productivity.

Model performance was rigorously assessed using the coefficient of determination (*R*^2^) and the mean absolute error (MAE) for both the training and test sets. Higher *R*^2^ and lower MAE values indicated better performance. Among the evaluated models, the Extreme Gradient Boosting (XGBoost) algorithm demonstrated the strongest predictive capacity for riverine Δ^14^C-DOC, achieving an *R*^2^ of 0.84 and an MAE of 52.63‰ for the training set, and an *R*^2^ of 0.73 and an MAE of 44.38‰ for the test set (Figure S4; Supplementary Table S4). The modest drop in performance from training to test sets (*R*^2^ decrease of 0.11) suggests limited overfitting, which was mitigated through regularization parameters (*λ* = 1, *α* = 0.5) and 10-fold cross-validation during hyperparameter tuning.

Geographic locations of river points were obtained from the Global Runoff Data Centre (GRDC, <https://mrb.grdc.bafg.de>). Using the 'Extract by Mask' tool in ArcToolbox, the GRDC ‘major rivers’ shapefile was applied to mask the global river dataset, resulting in 37,160 predicted Δ^14^C-DOC values extracted at GRDC river segment locations for major rivers worldwide.

To further validate the model performance, spatial comparisons between observed and predicted DOC concentration, δ^13^C-DOC, and Δ^14^C-DOC were conducted across the Yukon River Basin (Fig. S8). The results show good agreement between observations and predictions, demonstrating that the model successfully reproduces both the spatial patterns and statistical distributions of the observations.

### *Isotopic mixing model and isotopic end-member determinations*

EMMTE is a Visual Basic for Applications (VBA) tool for Microsoft Excel that performs source apportionment integrating a mass balance model with Monte Carlo random simulations [10]. Its performance demonstrated high consistency with the mixing Stable Isotope Analysis in R (MixSIAR), exhibiting superior goodness-of-prediction, a lower root mean square error (RMSE), and reduced relative deviation. In the EMMTE model, the mean isotopic values (δ^13^C and Δ^14^C) of the end-members were used as input, and the number of simulation iterations was set to 100,000. The increment, relative deviation, and minimum number of feasible solutions were configured as 1, 5, and 3, respectively [10]. Monte Carlo error, estimated from the standard deviation of contributions across iterations, averaged <3% for all end-members. We acknowledge several limitations: end-member values may vary spatially [11]; isotopic overlap between sources can increase attribution uncertainty, and; the model assumes conservative mixing, ignoring fractionation [10]. However, sensitivity analyses show that varying end-member values within ± 1 S.D. changes source contributions by <8%, indicating our global conclusions are robust.

End-members were selected followed two criteria: (*i*) each end-member should have constrained and literature-supported δ^13^C and Δ^14^C ranges, and; (*ii*) the end-members should exhibit limited isotopic overlap along δ^13^C and/or Δ^14^C axes. Potential end-members were first compiled from published datasets representing major DOC sources (Supplementary Table S5). Sources with similar isotopic values were grouped, and a single representative was retained from each group as the final end-member. For instance, C3 plants, contemporary terrestrial production, and terrestrial DOC clustered within a similar δ^13^C (approximately −28‰) and modern Δ^14^C range, and were therefore consolidated into a single terrestrial DOC end-member. Although C4 plants have distinct δ^13^C values, their contribution to global riverine DOC is comparatively minor and does not define a separate isotopic domain. Riverine autochthonous DOC, characterized by relatively enriched δ^13^C (approximately −20‰) and modern Δ^14^C, was retained as a distinct contemporary end-member. Holocene deposits exhibited depleted Δ^14^C signatures relative to modern sources and defined an intermediate-aged isotopic domain. Permafrost-derived and ancient DOC consistently showed extremely depleted Δ^14^C values, and were consolidated into a single ancient carbon end-member. Active layer soils and aged soil OC were not retained as independent end-members because they overlapped with both modern DOC and intermediate-aged carbon pools. Although soils are a major contributor to riverine DOC, soil-derived DOC spans a broad age spectrum, from recent plant inputs to deeper aged carbon, resulting in substantial overlap with both contemporary and old carbon pools.

To distinguish between contemporary and ancient carbon pools, we defined four isotopically distinct end-member sources based on a synthesis of published data (Supplementary Table S5). These end-members are not intended to capture site-specific biogeochemistry but to provide a globally-applicable framework for interpreting DOC sources based on our global observational dataset, serving as a practical framework to infer carbon source contributions at the global scale (Supplementary Table S5). The end-members are defined as follows:

- (*i*) Terrestrial DOC: Representing the youngest carbon pool with estimated average δ^13^C and Δ^14^C values of −28.6 ± 1.6‰ and 179.7 ± 65.9‰, respectively, based on data from tropical river systems [6]. This Δ^14^C value reflects rapid tropical carbon cycling and may be higher than in slower-turnover temperate or boreal systems.
- (*ii*) River autochthonous production: δ^13^C and Δ^14^C values were estimated as −19.8 ± 3‰ and 100 ± 50‰, respectively, reflecting the incorporation of modern atmospheric carbon and updating older estimates from Arctic systems [12].
- (*iii*) Holocene deposits: The mean Δ^14^C value of Holocene deposits was calculated as −567.5 ± 156.7‰ from exposures of peat and thermokarst in northern Siberia, while the mean δ^13^C values of Holocene deposits were calculated as −31 ± 1.0‰ [13, 14].
- (*iv*) Ancient (fossil) carbon. The estimated values for the δ^13^C and Δ^14^C ranges were determined to be −26.3 ± 0.7‰ and −954.8 ± 65.8‰, respectively, based on data obtained from Pleistocene Ice Complex deposits in northeastern Siberia [14].

These four end-members serve as bounding isotopic categories for estimating source contributions via end-member mixing analysis. We acknowledge that not all rivers will contain all source types, that there may be more than four potential sources, and that their relative abundance may vary regionally. We further acknowledge that fossil and permafrost-derived carbon can be isotopically similar (both highly depleted in ^14^C), meaning our “ancient carbon” end-member may encompass both petrogenic and Pleistocene permafrost sources where they co-occur. In high-elevation rivers, atmospheric deposition of industrial soot [8, 15] may contribute to the observed fossil signal, meaning the 6.7% global estimate may include both petrogenic and anthropogenic aerosol sources. However, this framework enables global-scale interpretation of DOC age patterns and source shifts, particularly in response to climate-driven changes such as permafrost thaw or enhanced primary productivity. These end-members provide a basis for quantifying the contributions of distinct carbon sources to riverine DOC.

*Geospatial analyses*

Grided maps of soil Δ^14^C-SOC were obtained from Zenodo (https://doi. org/10.5281/zenodo.3823612) at a 0.5° spatial resolution. Global riverine Δ^14^C-POC and riverine Δ^14^C-DIC were retrieved from Figshare (<https://doi.org/10.6084/m9.figshare.24268657>) and Zenodo (<https://doi.org/10.5281/zenodo.14989633>), respectively. Mean annual temperature (MAT) and mean annual precipitation (MAP) were sourced from WorldClim version 2.1 (<https://www.worldclim.org/data/worldclim21.html>) at 1 km resolution. Surface soil temperature, surface runoff, and subsurface runoff were derived from the ERA5-Land monthly aggregated reanalysis dataset provided by ECMWF (<https://cds.climate.copernicus.eu/>), and were accessed and processed using Google Earth Engine. To ensure spatial consistency across datasets, all variables except riverine Δ^14^C-POC were resampled to a common 0.5° spatial resolution, matching the coarsest input dataset. The matching of riverine Δ^14^C-POC with riverine Δ^14^C-DOC was achieved by aligning their sampling locations based on latitude and longitude coordinates. As riverine Δ^14^C-DIC data were only available from point observations, coordinate-based matching was similarly used to associate riverine Δ^14^C-DIC values with riverine Δ^14^C-DOC.


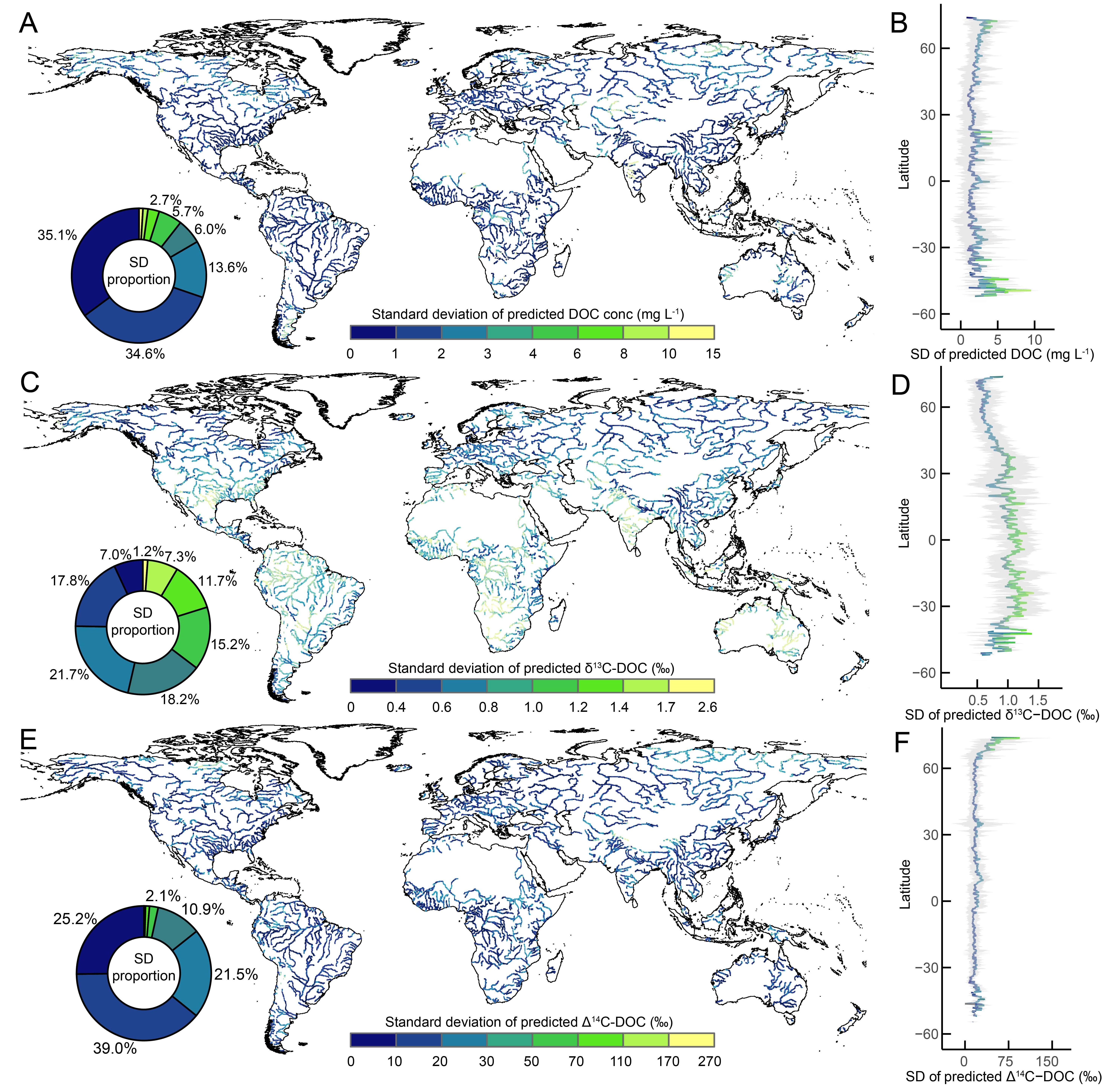
Fig. S1. Global distributions and latitudinal patterns of prediction uncertainty for riverine DOC concentrations, δ^13^C-DOC, and Δ^14^C-DOC values. The global spatial distribution and latitudinal patterns of predicted uncertainties in riverine (A−B) DOC concentrations, (C−D) δ^13^C-DOC, and (E−F) Δ^14^C-DOC values are assessed using the standard deviation (SD). The map in panels (A, C, E) includes 37,160 predicted values, aligning with the major river datasets from GRDC (<https://mrb.grdc.bafg.de>). The colored lines in panels (B), (D) and (F) illustrate the average SD values of riverine DOC concentrations, δ^13^C-DOC, and Δ^14^C-DOC values across latitudes, with grey shading represents ±1 standard deviation. Model uncertainty for Δ^14^C-DOC averaged 18.8 ± 15.9‰, with highest predictive errors in data-sparse Arctic regions.





Fig. S2. Key environmental variables influencing riverine dissolved organic carbon (DOC) concentrations. (A) Pearson correlation coefficients between various environmental variables and riverine DOC concentrations, quantifying the strength and direction of these associations. (B) The percentage increase in mean square error (MSE) illustrates the importance of each environmental variable, as determined by the random forest approach. A higher percentage increase in MSE signifies a greater contribution of the corresponding variable to the predictive model. The performance of feature-selected random forest model applied to the training set (C) and the testing set (D). *n* represents the number of the samples, with key metrics including the coefficient of determination (*R*²) and mean absolute error (MAE) shown. (E−M) Partial dependence plots show the marginal effect of the most important environmental variables on DOC concentrations, selected based on Pearson correlation analysis and the random forest method. DOC concentration generally increases with available water content, decreases with elevation, and shows positive association with soil C/N ratio. Note that y-axis ranges differ among panels (E−M) to best reflect the variability within each individual predictor.





Fig. S3. Key environmental variables influencing δ^13^C values of riverine dissolved organic carbon (DOC). (A) Pearson correlation coefficients between various environmental variables and riverine δ^13^C-DOC. (B) The percentage increase in mean square error (MSE) illustrates the importance of each environmental variable, as determined by the random forest approach. The performance of feature-selected XGBoost model applied to the training set (C) and the testing set (D). (E−N) Partial dependence plots show the marginal effect of the most important environmental variables on δ^13^C-DOC, selected based on Pearson correlation analysis and the random forest method. δ^13^C-DOC generally increases (becomes less negative) with increasing elevation. Note that y-axis ranges differ among panels (E−N) to best reflect the variability within each individual predictor.





Fig. S4. Key environmental variables influencing Δ^14^C values of riverine dissolved organic carbon (DOC). (A) Pearson correlation coefficients between various environmental variables and riverine Δ^14^C-DOC. (B) The percentage increase in mean square error (MSE) illustrates the importance of each environmental variable, as determined by the random forest approach. The performance of feature-selected XGBoost model applied to the training set (C) and the testing set (D). (E−N) Partial dependence plots show the marginal effect of the most important environmental variables on Δ^14^C-DOC, selected based on Pearson correlation analysis and the random forest method. Δ^14^C-DOC generally increases (becomes less negative) with decreasing elevation. Note that y-axis ranges differ among panels (E−N) to best reflect the variability within each individual predictor.


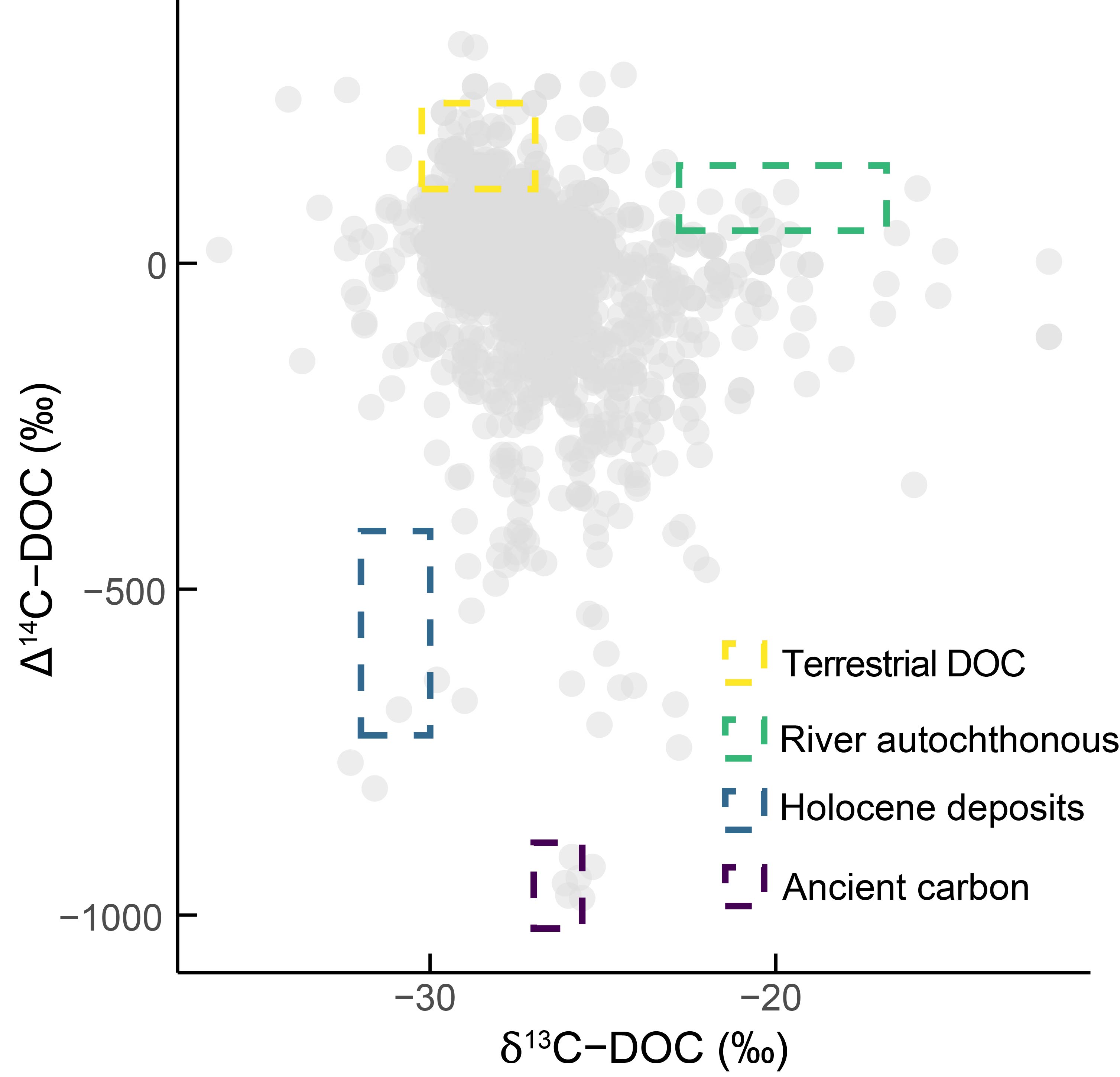


Fig. S5. Characterization of global riverine dissolved organic carbon (DOC) pools using a dual-isotope mixing model. Distributions of δ^13^C and Δ^14^C values, along with global riverine carbon pools, were characterized using end-member mixing analysis based on natural abundance δ^13^C and Δ^14^C values of DOC. See Table S5 for a complete description of end-member sources and their respective isotopic ranges.


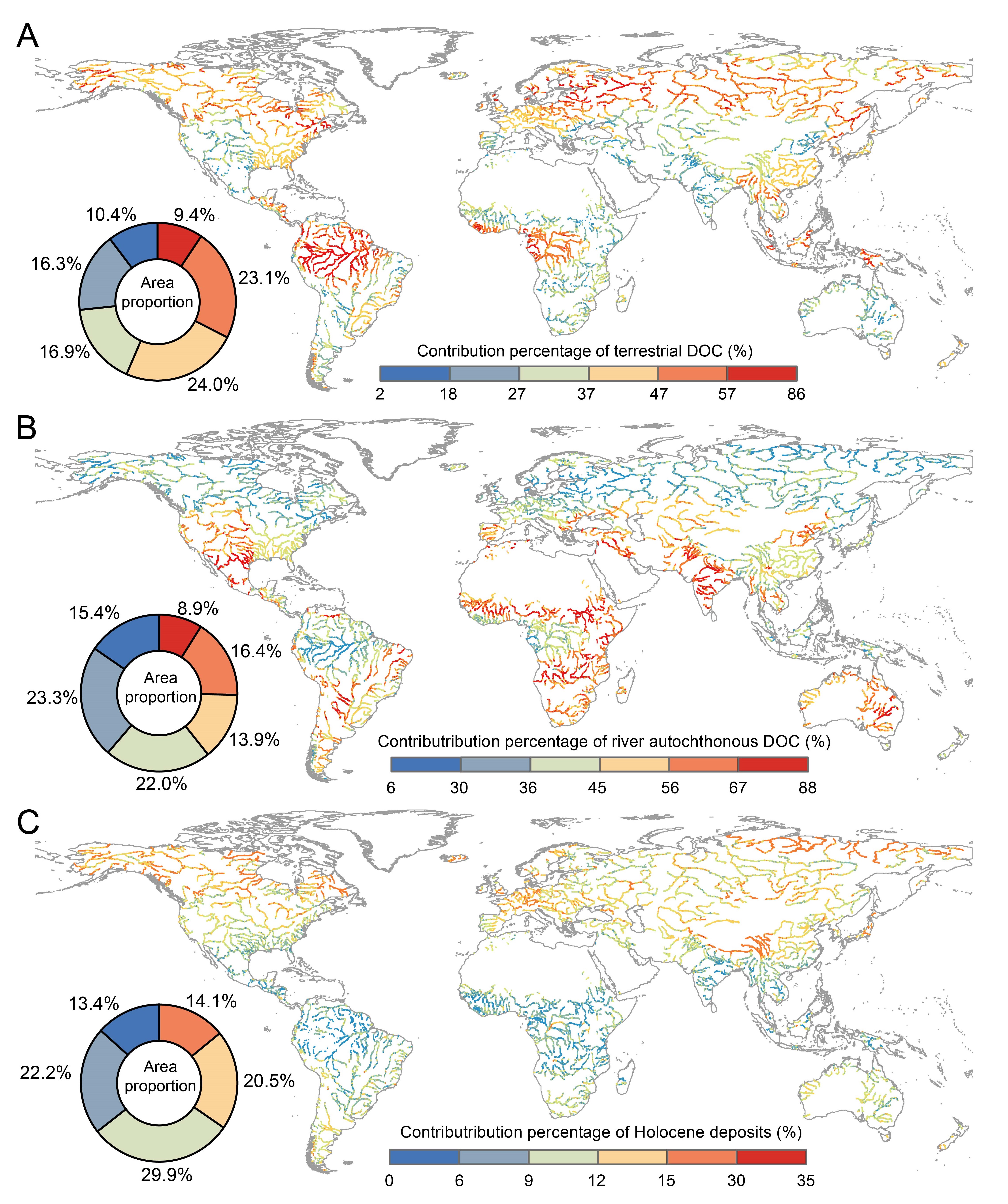


Fig. S6. Global distribution of dissolved organic carbon (DOC) source contributions. Spatial distribution of the predicted proportional contribution to the riverine DOC pool from (A) terrestrial sources, (B) aquatic autochthonous production, and (C) Holocene deposits.


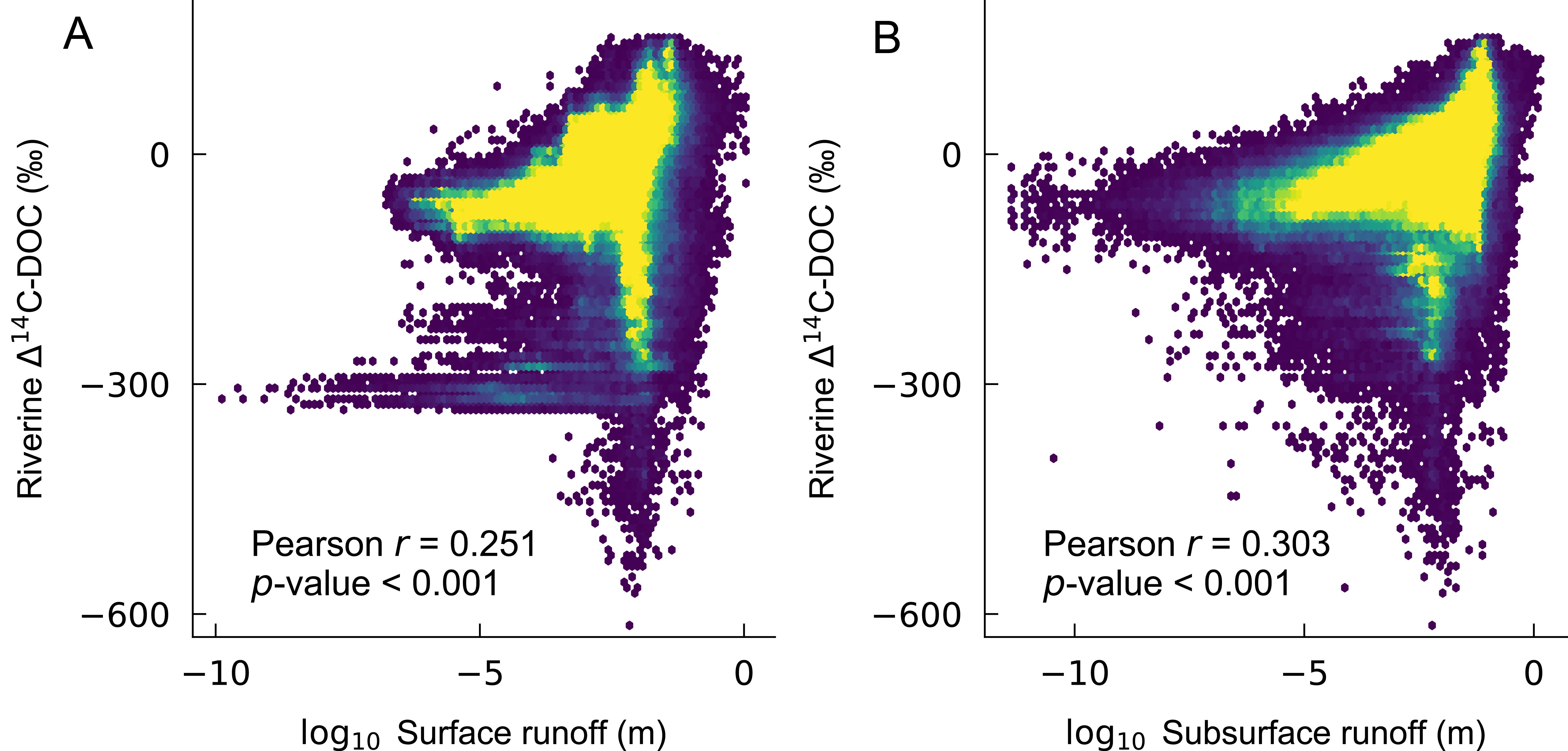


Fig. S7. Relationship between riverine Δ^14^C-DOC and hydrological runoff. Association of Δ^14^C-DOC with (A) surface runoff and (B) subsurface runoff. All correlations are derived from globally distributed data points and are statistically significant (*p* < 0.001).


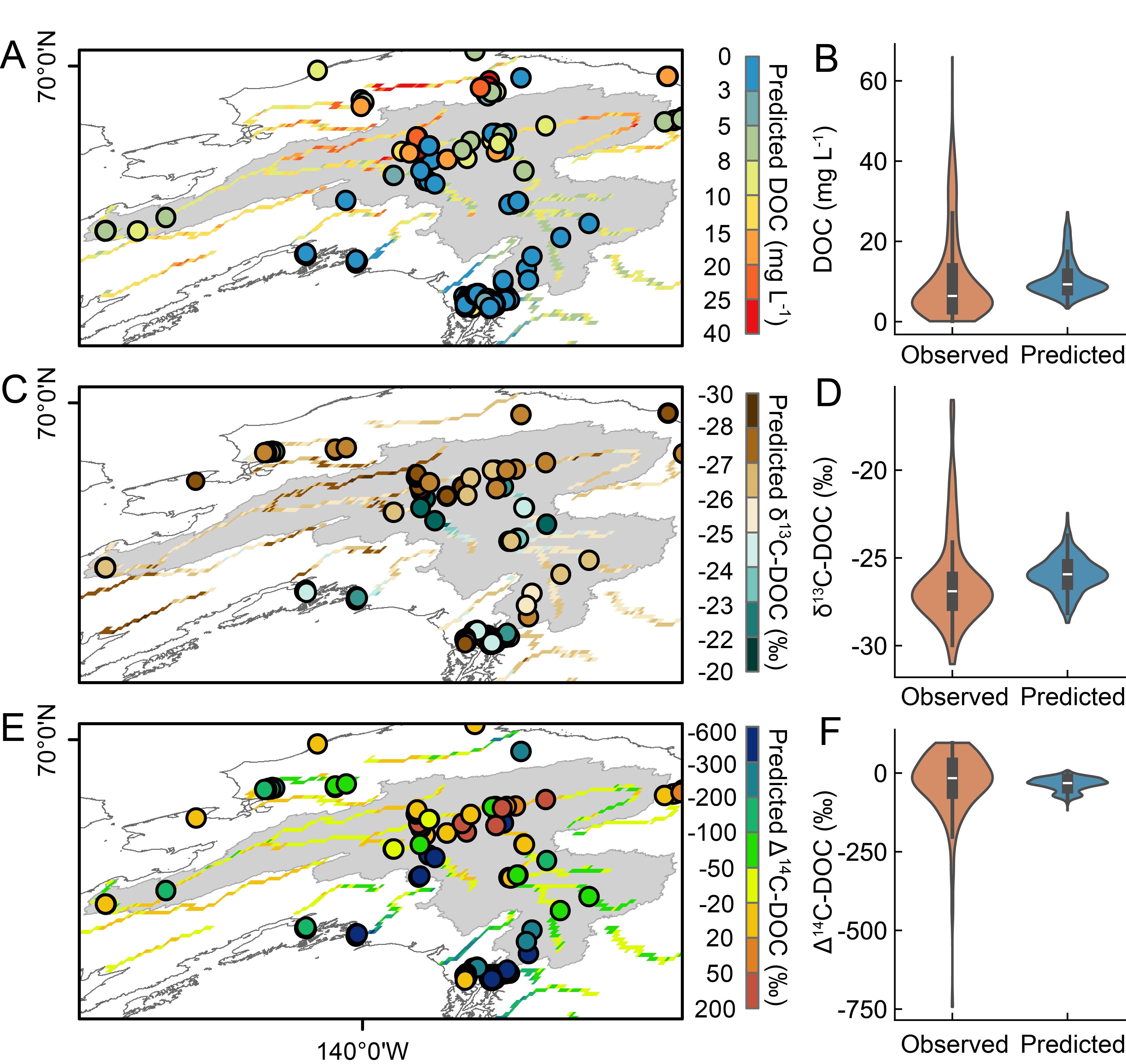


Fig. S8. Distribution of observed and predicted riverine DOC concentration, δ^13^C-DOC and Δ^14^C-DOC in the Yukon River Basin. Panels (A), (C) and (E) show 357 DOC concentration measurements, 162 δ^13^C-DOC observations and 200 Δ^14^C-DOC observations respectively, together with the corresponding predicted variables in Yukon River. In these panels, colored dots represent observational data, while the colored pixels indicate the corresponding model-predicted values. Panels (B), (D) and (F) present comparisons between observed and predicted DOC concentration, δ^13^C-DOC and Δ^14^C-DOC for the Yukon River Basin.

Table S1. Environmental variables used as training data for dissolved organic carbon (DOC) concentrations, δ^13^C and Δ^14^C values. These variables were systematically selected to represent diverse aspects of Earth's surface properties and processes.

| **Variable Names** | **Layer Group** | **Source databases** |
| --- | --- | --- |
| Available water content | Soil Property | Harmonized World Soil Database v 2.0  (<https://www.fao.org/soils-portal/data-hub/soil-maps-and-databases/harmonized-world-soil-database-v20/en/>) |
| C/N ratio | Soil Property | Harmonized World Soil Database v 2.0  (<https://www.fao.org/soils-portal/data-hub/soil-maps-and-databases/harmonized-world-soil-database-v20/en/>) |
| Silt & Clay | Soil Property | Harmonized World Soil Database v 2.0  (<https://www.fao.org/soils-portal/data-hub/soil-maps-and-databases/harmonized-world-soil-database-v20/en/>) |
| Sand | Soil Property | Harmonized World Soil Database v 2.0  (<https://www.fao.org/soils-portal/data-hub/soil-maps-and-databases/harmonized-world-soil-database-v20/en/>) |
| Course | Soil Property | Harmonized World Soil Database v 2.0  (<https://www.fao.org/soils-portal/data-hub/soil-maps-and-databases/harmonized-world-soil-database-v20/en/>) |
| Total nitrogen | Soil Property | Harmonized World Soil Database v 2.0  (<https://www.fao.org/soils-portal/data-hub/soil-maps-and-databases/harmonized-world-soil-database-v20/en/>) |
| Soil water retention | Soil Property | Harmonized World Soil Database v 2.0  (<https://www.fao.org/soils-portal/data-hub/soil-maps-and-databases/harmonized-world-soil-database-v20/en/>) |
| Surface volumetric soil water | Soil Property | ERA5-Land Monthly Aggregated - ECMWF Climate Reanalysis  ([https://developers.google.com/earth‑engine/datasets/catalog/ECMWF_ERA5_LAND_MONTHLY_AGGR](https://developers.google.com/earth-engine/datasets/catalog/ECMWF_ERA5_LAND_MONTHLY_AGGR) ) |
| Soil moisture | Soil Property | Terra Climate  ([https://developers.google.com/earth‑engine/datasets/catalog/IDAHO_EPSCOR_TERRACLIMATE#bands](https://developers.google.com/earth-engine/datasets/catalog/IDAHO_EPSCOR_TERRACLIMATE#bands) ) |
| Palmer drought severity index | Soil Property | Terra Climate  ([https://developers.google.com/earth‑engine/datasets/catalog/IDAHO_EPSCOR_TERRACLIMATE#bands](https://developers.google.com/earth-engine/datasets/catalog/IDAHO_EPSCOR_TERRACLIMATE#bands) ) |
| Surface soil temperature | Climate | ERA5-Land Monthly Aggregated - ECMWF Climate Reanalysis  ([https://developers.google.com/earth‑engine/datasets/catalog/ECMWF_ERA5_LAND_MONTHLY_AGGR](https://developers.google.com/earth-engine/datasets/catalog/ECMWF_ERA5_LAND_MONTHLY_AGGR) ) |
| Climate water deficit | Climate | Terra Climate  ([https://developers.google.com/earth‑engine/datasets/catalog/IDAHO_EPSCOR_TERRACLIMATE#bands](https://developers.google.com/earth-engine/datasets/catalog/IDAHO_EPSCOR_TERRACLIMATE#bands) ) |
| Wind speed | Climate | Terra Climate  ([https://developers.google.com/earth‑engine/datasets/catalog/IDAHO_EPSCOR_TERRACLIMATE#bands](https://developers.google.com/earth-engine/datasets/catalog/IDAHO_EPSCOR_TERRACLIMATE#bands) ) |
| Reference evapotranspiration | Climate | Terra Climate  ([https://developers.google.com/earth‑engine/datasets/catalog/IDAHO_EPSCOR_TERRACLIMATE#bands](https://developers.google.com/earth-engine/datasets/catalog/IDAHO_EPSCOR_TERRACLIMATE#bands) ) |
| Actual evapotranspiration | Climate | Terra Climate  ([https://developers.google.com/earth‑engine/datasets/catalog/IDAHO_EPSCOR_TERRACLIMATE#bands](https://developers.google.com/earth-engine/datasets/catalog/IDAHO_EPSCOR_TERRACLIMATE#bands) ) |
| Surface shortwave radiation | Climate | Terra Climate  ([https://developers.google.com/earth‑engine/datasets/catalog/IDAHO_EPSCOR_TERRACLIMATE#bands](https://developers.google.com/earth-engine/datasets/catalog/IDAHO_EPSCOR_TERRACLIMATE#bands) ) |
| Snow water equivalent | Climate | Terra Climate  ([https://developers.google.com/earth‑engine/datasets/catalog/IDAHO_EPSCOR_TERRACLIMATE#bands](https://developers.google.com/earth-engine/datasets/catalog/IDAHO_EPSCOR_TERRACLIMATE#bands) ) |
| Vapor pressure deficit | Climate | Terra Climate  ([https://developers.google.com/earth‑engine/datasets/catalog/IDAHO_EPSCOR_TERRACLIMATE#bands](https://developers.google.com/earth-engine/datasets/catalog/IDAHO_EPSCOR_TERRACLIMATE#bands) ) |
| Total evaporation sum | Climate | ERA5-Land Monthly Aggregated - ECMWF Climate Reanalysis  ([https://developers.google.com/earth‑engine/datasets/catalog/ECMWF_ERA5_LAND_MONTHLY_AGGR](https://developers.google.com/earth-engine/datasets/catalog/ECMWF_ERA5_LAND_MONTHLY_AGGR) ) |
| V component of wind at 10m | Climate | ERA5-Land Monthly Aggregated - ECMWF Climate Reanalysis  ([https://developers.google.com/earth‑engine/datasets/catalog/ECMWF_ERA5_LAND_MONTHLY_AGGR](https://developers.google.com/earth-engine/datasets/catalog/ECMWF_ERA5_LAND_MONTHLY_AGGR)) |
| Surface runoff sum | Climate | ERA5-Land Monthly Aggregated - ECMWF Climate Reanalysis  ([https://developers.google.com/earth‑engine/datasets/catalog/ECMWF_ERA5_LAND_MONTHLY_AGGR](https://developers.google.com/earth-engine/datasets/catalog/ECMWF_ERA5_LAND_MONTHLY_AGGR)) |
| Runoff sum | Climate | ERA5-Land Monthly Aggregated - ECMWF Climate Reanalysis  ([https://developers.google.com/earth‑engine/datasets/catalog/ECMWF_ERA5_LAND_MONTHLY_AGGR](https://developers.google.com/earth-engine/datasets/catalog/ECMWF_ERA5_LAND_MONTHLY_AGGR)) |
| Total precipitation sum | Climate | ERA5-Land Monthly Aggregated - ECMWF Climate Reanalysis  ([https://developers.google.com/earth‑engine/datasets/catalog/ECMWF_ERA5_LAND_MONTHLY_AGGR](https://developers.google.com/earth-engine/datasets/catalog/ECMWF_ERA5_LAND_MONTHLY_AGGR)) |
| Mean annual temperature | Climate | WorldClim v 2.1  (<https://www.worldclim.org/data/worldclim21.html>) |
| Mean annual precipitation | Climate | WorldClim v 2.1  (<https://www.worldclim.org/data/worldclim21.html>) |
| Gross primary productivity | Primary productivity | MOD17A3HGF.061([https://developers.google.com/earth‑engine/datasets/catalog/MODIS_061_MOD17A3HGF#bands](https://developers.google.com/earth-engine/datasets/catalog/MODIS_061_MOD17A3HGF#bands) ) |
| Net primary productivity | Primary productivity | MOD17A3HGF.061([https://developers.google.com/earth‑engine/datasets/catalog/MODIS_061_MOD17A3HGF#bands](https://developers.google.com/earth-engine/datasets/catalog/MODIS_061_MOD17A3HGF#bands) ) |
| Total soil respiration | Primary productivity | (<http://cse.ffpri.affrc.go.jp/shojih/data/index.html>) |
| Leaf area index of low vegetation | Primary productivity | ERA5-Land Monthly Aggregated - ECMWF Climate Reanalysis  ([https://developers.google.com/earth‑engine/datasets/catalog/ECMWF_ERA5_LAND_MONTHLY_AGGR](https://developers.google.com/earth-engine/datasets/catalog/ECMWF_ERA5_LAND_MONTHLY_AGGR)) |
| Leaf area index of high vegetation | Primary productivity | ERA5-Land Monthly Aggregated - ECMWF Climate Reanalysis  ([https://developers.google.com/earth‑engine/datasets/catalog/ECMWF_ERA5_LAND_MONTHLY_AGGR](https://developers.google.com/earth-engine/datasets/catalog/ECMWF_ERA5_LAND_MONTHLY_AGGR)) |
| Elevation | Geomorphology | GMTED2010 (<http://www.earthenv.org/topography>) |
| Slope | Geomorphology | GMTED2010 (<http://www.earthenv.org/topography>) |
| R factor | Geomorphology | GloSEM(https://esdac.jrc.ec.europa.eu/content/global-soil-erosion) |
| C factor | Geomorphology | GloSEM(https://esdac.jrc.ec.europa.eu/content/global-soil-erosion) |
| K factor | Geomorphology | GloSEM(https://esdac.jrc.ec.europa.eu/content/global-soil-erosion) |
| LS factor | Geomorphology | GloSEM(https://esdac.jrc.ec.europa.eu/content/global-soil-erosion) |
| Soil loss | Geomorphology | GloSEM(https://esdac.jrc.ec.europa.eu/content/global-soil-erosion) |
| Human development index | Anthropogenic perturbations | Dryad (<https://datadryad.org/stash/dataset/doi:10.5061/dryad.dk1j0> |
| Gross domestic product | Anthropogenic perturbations | Dryad (<https://datadryad.org/stash/dataset/doi:10.5061/dryad.dk1j0> |
| Average DNB radiance | Anthropogenic perturbations | VIIRS Nighttime Day/Night Band Composites Version 1  ([https://developers.google.com/earth‑engine/datasets/catalog/NOAA_VIIRS_DNB_MONTHLY_V1_VCMCFG](https://developers.google.com/earth-engine/datasets/catalog/NOAA_VIIRS_DNB_MONTHLY_V1_VCMCFG)) |
| Population density | Anthropogenic perturbations | GPWv411([https://developers.google.com/earth‑engine/datasets/catalog/CIESIN_GPWv411_GPW_Population_Density](https://developers.google.com/earth-engine/datasets/catalog/CIESIN_GPWv411_GPW_Population_Density)) |

**Table S2.** **Statistical model performance for predicted riverine dissolved organic carbon (DOC) concentrations.**

| Model | | Generalized variables | | | | Feature selection | | | |
| --- | --- | --- | --- | --- | --- | --- | --- | --- | --- |
| Model selection | | Training | | Test | | Training | | Test | |
|  |  | R^2^ | MAE | R^2^ | MAE | R^2^ | MAE | R^2^ | MAE |
| Linear | Linear | 0.27 | 3.825 | 0.27 | 3.947 | 0.12 | 4.056 | 0.10 | 4.164 |
|  | Lasso | 0.19 | 3.892 | 0.17 | 4.055 | 0.11 | 4.087 | 0.08 | 4.187 |
|  | Ridge | 0.27 | 3.814 | 0.27 | 3.928 | 0.12 | 4.607 | 0.10 | 4.169 |
| Decision Tree | | 0.68 | 2.432 | 0.64 | 2.731 | 0.66 | 2.419 | 0.63 | 2.704 |
| K Neighbors | | 0.68 | 2.377 | 0.61 | 2.608 | 0.65 | 2.474 | 0.62 | 2.806 |
| Extra Tree | | 0.71 | 2.231 | 0.62 | 2.575 | 0.49 | 3.214 | 0.46 | 2.975 |
| Adaboost | | 0.16 | 5.609 | 0.18 | 5.720 | 0.17 | 5.830 | 0.12 | 5.644 |
| GBDT | | 0.74 | 2.302 | 0.69 | 2.570 | 0.69 | 2.500 | 0.67 | 2.705 |
| XGBoost | | 0.82 | 1.702 | 0.71 | 2.339 | 0.81 | 1.769 | 0.71 | 2.418 |
| CatBoost | | 0.62 | 2.810 | 0.61 | 3.018 | 0.60 | 2.956 | 0.59 | 3.053 |
| LightGBM | | 0.71 | 2.351 | 0.66 | 2.632 | 0.64 | 2.613 | 0.62 | 2.813 |
| Random Forest | | **0.79** | 1.868 | 0.70 | 2.376 | **0.78** | 1.919 | **0.72** | 2.390 |
| Bagging | | 0.78 | 1.903 | 0.69 | 2.476 | 0.77 | 1.949 | 0.69 | 2.568 |

**Table S3.** **Statistical model performance for predicted δ^13^C values in riverine dissolved organic carbon (DOC).**

| Model | | Generalized variables | | | | Feature selection | | | |
| --- | --- | --- | --- | --- | --- | --- | --- | --- | --- |
| Model selection | | Training | | Test | | Training | | Test | |
|  |  | R^2^ | MAE | R^2^ | MAE | R^2^ | MAE | R^2^ | MAE |
| Linear | Linear | 0.45 | 1.262 | 0.43 | 1.263 | 0.35 | 1.395 | 0.33 | 1.429 |
|  | Lasso | 0.35 | 1.453 | 0.31 | 1.442 | 0.29 | 1.481 | 0.28 | 1.489 |
|  | Ridge | 0.44 | 1.278 | 0.42 | 1.283 | 0.35 | 1.395 | 0.33 | 1.429 |
| Decision Tree | | 0.61 | 0.971 | 0.47 | 1.205 | 0.55 | 1.109 | 0.50 | 1.139 |
| K Neighbors | | 0.53 | 1.037 | 0.48 | 1.169 | 0.56 | 1.086 | 0.50 | 1.166 |
| Extra Tree | | 0.52 | 1.084 | 0.47 | 1.222 | 0.53 | 1.164 | 0.52 | 1.173 |
| Adaboost | | 0.19 | 1.574 | 0.13 | 1.637 | 0.28 | 1.609 | 0.28 | 1.583 |
| GBDT | | 0.64 | 0.916 | 0.57 | 1.101 | 0.63 | 0.993 | 0.60 | 1.050 |
| XGBoost | | **0.68** | 0.825 | 0.56 | 1.093 | **0.68** | 0.895 | **0.62** | 1.013 |
| CatBoost | | 0.58 | 1.044 | 0.52 | 1.182 | 0.56 | 1.108 | 0.53 | 1.160 |
| LightGBM | | 0.61 | 0.966 | 0.56 | 1.100 | 0.59 | 1.047 | 0.59 | 1.048 |
| Random Forest | | 0.66 | 0.813 | 0.54 | 1.092 | 0.66 | 0.858 | 0.63 | 0.994 |
| Bagging | | 0.65 | 0.859 | 0.54 | 1.127 | 0.66 | 0.904 | 0.60 | 1.029 |

**Table S4.** **Statistical model performance for predicted Δ^14^C values in riverine dissolved organic carbon (DOC).**

| Model | | Generalized variables | | | | Feature selection | | | |
| --- | --- | --- | --- | --- | --- | --- | --- | --- | --- |
| Model selection | | Training | | Test | | Training | | Test | |
|  |  | R^2^ | MAE | R^2^ | MAE | R^2^ | MAE | R^2^ | MAE |
| Linear | Linear | 0.39 | 73.703 | 0.37 | 78.867 | 0.26 | 84.654 | 0.25 | 84.288 |
|  | Lasso | 0.38 | 74.153 | 0.36 | 78.780 | 0.25 | 84.963 | 0.24 | 84.304 |
|  | Ridge | 0.38 | 74.131 | 0.36 | 79.107 | 0.26 | 84.730 | 0.25 | 84.098 |
| Decision Tree | | 0.75 | 46.305 | 0.50 | 65.543 | 0.67 | 53.764 | 0.60 | 59.970 |
| K Neighbors | | 0.69 | 49.143 | 0.45 | 68.594 | 0.66 | 52.431 | 0.53 | 63.476 |
| Extra Tree | | 0.68 | 53.553 | 0.54 | 65.185 | 0.63 | 58.668 | 0.47 | 63.770 |
| Adaboost | | 0.51 | 86.148 | 0.42 | 91.651 | 0.35 | 102.09 | 0.30 | 101.53 |
| GBDT | | 0.81 | 44.040 | 0.66 | 56.638 | 0.75 | 50.531 | 0.67 | 55.508 |
| XGBoost | | **0.83** | 42.328 | 0.66 | 56.534 | **0.84** | 44.379 | **0.73** | 52.632 |
| CatBoost | | 0.71 | 54.713 | 0.50 | 67.962 | 0.64 | 61.555 | 0.64 | 62.024 |
| LightGBM | | 0.78 | 46.423 | 0.63 | 58.385 | 0.72 | 52.119 | 0.67 | 56.144 |
| Random Forest | | 0.76 | 48.703 | 0.61 | 59.997 | 0.73 | 52.636 | 0.64 | 57.681 |
| Bagging | | 0.69 | 30.515 | 0.34 | 69.882 | 0.73 | 29.893 | 0.44 | 62.451 |

**Table S5.** **Compiled carbon isotopic values for global riverine dissolved organic carbon (DOC) with the bolded is the final selected end member.** Note that the terrestrial DOC end-member is derived from measurements in tropical river systems where high primary productivity and rapid carbon turnover yield exceptionally modern Δ^14^C signatures. End-members were selected to represent the full isotopic range of potential DOC sources globally.

| **Source** | **Δ^14^C-DOC** | **δ^13^C-DOC** | **References** |
| --- | --- | --- | --- |
| **Terrestrial DOC** | **179.72 (± 65.89)** | **–28.6 (± 1.64)** | **Ref. [7]** |
| C3 plants | 120 (± 60) | –28 (± 4) | Ref. [11] |
| C4 plants | 120 (± 60) | –11.5 (± 1.5) | Ref. [11] |
| Contemporary | 71.6 (± 170) | –28.5 (± 2) | Ref. [12] |
| Terrestrial primary production | 97 (± 124.8) | –27.7 (± 1.3) | Ref. [14] |
| **River autochthonous DOC** | **100 (± 50)** | **–19.8 (± 3)** | **Ref. [11], Ref. [12]** |
| Active layer | –197.5 (± 148.3) | –26.4 (± 0.8) | Ref. [14] |
| Aged soil OC | –315.37 (± 84.27) | –23.76 (± 0.09) | Ref. [7] |
| **Holocene deposits** | **–567.5 (± 156.7)** | **–31 (± 1)** | **Ref. [13], Ref. [14]** |
| Permafrost | –945.83 (± 22.43) | –25.76 (± 0.26) | Ref. [16] |
| Permafrost | –940 (± 84) | –26.3 (± 1.3) | Ref. [12] |
| **Ancient DOC** | **–954.8 (± 65.8)** | **–26.3 (± 0.7)** | **Ref. [14]** |

**References**

1. McNichol AP, Aluwihare LI. The power of radiocarbon in biogeochemical studies of the marine carbon cycle: Insights from studies of dissolved and particulate organic carbon (DOC and POC). *Chem Rev*. 2007; **107**(2): 443-466.

2. Eglinton TI, Galy VV, Hemingway JD *et al.* Climate control on terrestrial biospheric carbon turnover. *Proc Natl Acad Sci USA*. 2021; **118**(8): e2011585118.

3. Butman DE, Wilson HF, Barnes RT *et al.* Increased mobilization of aged carbon to rivers by human disturbance. *Nat Geosci*. 2015; **8**(2): 112-116.

4. Galy V, Eglinton T. Protracted storage of biospheric carbon in the Ganges–Brahmaputra basin. *Nat Geosci*. 2011; **4**(12): 843-847.

5. Schefuß E, Eglinton TI, Spencer-Jones CL *et al.* Hydrologic control of carbon cycling and aged carbon discharge in the Congo River basin. *Nat Geosci*. 2016; **9**(9): 687-690.

6. Mayorga E, Aufdenkampe AK, Masiello CA *et al.* Young organic matter as a source of carbon dioxide outgassing from Amazonian rivers. *Nature*. 2005; **436**(7050): 538-541.

7. Drake TW, Van Oost K, Barthel M *et al.* Mobilization of aged and biolabile soil carbon by tropical deforestation. *Nat Geosci*. 2019; **12**(7): 541-546.

8. Stubbins A, Hood E, Raymond PA *et al.* Anthropogenic aerosols as a source of ancient dissolved organic matter in glaciers. *Nat Geosci*. 2012; **5**: 198-201.

9. Xiang D, Wang G, Tian J *et al.* Global patterns and edaphic-climatic controls of soil carbon decomposition kinetics predicted from incubation experiments. *Nat Commun*. 2023; **14**(1): 2171.

10. Cao X, He W, He W *et al.* EMMTE: An Excel VBA tool for source apportionment of nitrate based on the stable isotope mixing model. *Sci Total Environ*. 2023; **868**: 161728.

11. Marwick TR, Tamooh F, Teodoru CR *et al.* The age of river‐transported carbon: A global perspective. *Global Biogeochem Cy*. 2015; **29**(2): 122-137.

12. Mann PJ, Eglinton TI, McIntyre CP *et al.* Utilization of ancient permafrost carbon in headwaters of arctic fluvial networks. *Nat Commun*. 2015; **6**: 7856.

13. Godfrey LV, Herrera C, Burr GS *et al.* δ^13^C and ^14^C activity of groundwater DOC and DIC in the volcanically active and arid Loa Basin of northern Chile. *J Hydrol*. 2021; **595**: 125987.

14. Wild B, Andersson A, Broder L *et al.* Rivers across the Siberian Arctic unearth the patterns of carbon release from thawing permafrost. *Proc Natl Acad Sci USA*. 2019; **116**(21): 10280-10285.

15. Holt AD, McKenna AM, Kellerman AM *et al.* Gradients of deposition and in situ production drive global glacier organic matter composition. *Global Biogeochem Cycle*. 2024; **38**(9): e2024GB008212.

16. Vonk JE, Sanchez-Garcia L, van Dongen BE *et al.* Activation of old carbon by erosion of coastal and subsea permafrost in Arctic Siberia. *Nature*. 2012; **489**(7414): 137-140.
